# Natural Sweeteners: The Relevance of Food Naturalness for Consumers, Food Security Aspects, Sustainability and Health Impacts

**DOI:** 10.3390/ijerph17176285

**Published:** 2020-08-28

**Authors:** Ariana Saraiva, Conrado Carrascosa, Dele Raheem, Fernando Ramos, António Raposo

**Affiliations:** 1 Department of Animal Pathology and Production, Bromatology and Food Technology, Faculty of Veterinary, Universidad de Las Palmas de Gran Canaria, Trasmontaña s/n, 35413 Arucas, Spain; ariana_23@outlook.pt (A.S.); conrado.carrascosa@ulpgc.es (C.C.); 2 Northern Institute for Environmental and Minority Law (NIEM), Arctic Centre, University of Lapland, 96101 Rovaniemi, Lapland, Finland; Bamidele.Raheem@ulapland.fi; 3 Pharmacy Faculty, University of Coimbra, Azinhaga de Santa Comba, 3000-548 Coimbra, Portugal; framos@ff.uc.pt; 4 REQUIMTE/LAQV, R. D. Manuel II, Apartado 55142 Oporto, Portugal; 5 Department for Management of Science and Technology Development, Ton Duc Thang University, Ho Chi Minh City, Vietnam; 6 Faculty of Environment and Labour Safety, Ton Duc Thang University, Ho Chi Minh City, Vietnam

**Keywords:** consumer’s perceptions and attitudes, food industry, food security, health impacts, natural food products, natural sweeteners, sustainability

## Abstract

At a moment when the population is increasingly aware and involved in what it eats, both consumers and the food sector are showing more interest in natural foods. This review work discusses, addresses and provides details of the most important aspects of consumer’s perceptions of and attitudes to natural foods and in-depth research into natural sweeteners. It also includes issues about their use and development as regards health impacts, food security and sustainability. In line with our main research outcome, we can assume that consumers are very keen on choosing foods with clean labelling, natural ingredients, preferably with other functional properties, without the loss of taste. In response to such a phenomenon, the food industry offers consumers alternative natural sweeteners with the advantage of added health benefits. It is noteworthy that Nature is a superb source of desirable substances, and many have a sweet taste, and many still need to be studied. Finally, we must stress that being natural does not necessarily guarantee market success.

## 1. Introduction

In the 20th century, developed countries resolved the lack of food security with a major contribution from agri-food industrialisation [1,2,3]. Food processing has played a vital role in prolonging food products’ shelf life, mitigating food losses and reducing waste and in enhancing the production of nutrients and their availability [4,5]. Yet day-to-day consumer perceptions rely on other factors apart from these achievements. In modern societies, more globalised markets and more manufacturing efforts made in the food chain have led to knowledge gaps and a perceived separation between local manufacturers and citizens (e.g., how foods are produced, where they are produced, etc.) [6,7]. Consumers are gradually becoming more aware to natural ingredients, while the growing importance of naturalness among consumers has meant key implications for the food industry [8]. This could well have implications for not only developing and selling food, but also for the increase in emerging food technologies. It is possible that those food products not perceived as natural are not accepted by lots of consumers in the majority of countries.

The demand for zero-calorie and naturally derived sweeteners has dramatically grown in the last decade because consumers are more mindful of their health [9]. For decades sweeteners have been used to make food more flavourful and to attract consumers. They were first adopted because of high-calorie sugar-to-diet ratio, and this favoured obesity in the general population, which became widespread in infants and children [10]. Thus, a low-calorie sweetener, saccharine, became available in the 1980s. As this sweetener was so popular, others followed, including cyclamates, aspartame and acesulfame K, which are the most widespread. Sweeteners have long-since been the object of controversies and conflicts over the years, which have included allegations of liver and bladder toxicity, carcinogenicity, foetus malformations, along with other dangers [11]. Whereas all these allegations have been investigated, sweeteners were considered safe [12], although some loss of consumer trust remains as some are not permitted in the USA, while others are allowed in the EU (e.g., cyclamate and cyclamic acid), but are not permitted in the USA (under E 952). Hence the need for natural substitutes is crucial [13]. Natural sweeteners and synthetic sweeteners have the same purpose: to act as a sweet flavour while fewer or no calories at all to diet. Natural sweeteners can be classified as two categories: high-potency sweeteners and bulk sweeteners. The former’s potency is greater than the sweetness of one sucrose molecule. The latter’s potency is the equivalent to one sucrose molecule, or less, with sucrose being the international standard for sweetness.

The main aims of this review were to provide details of and understand consumers perceptions and attitudes to natural food products, and to study in-depth natural sweeteners, mostly aspects related to their use and production in health impacts, food security and sustainability terms. Special attention was given to sweeteners, which are unanimously considered in the literature to have a good taste, high solubility and high stability, be safe with an acceptable cost-on-use, namely, erythritol glycyrrhizin, tagatose, steviol glycosides and thaumatin.

## 2. Consumer Perceptions of and Attitudes to Natural Food Products

Humans are inherently connected to natural objects [14], so it should come as no surprise that most humans have clearly preferred natural foods in the last few decades [8,15]. The findings of the Nielsen Global Health and Wellness Survey [16], which was conducted in 60 countries and involved 30,000 consumers, revealed that the most essential food characteristics are naturalness, freshness and minimal processing. The findings of the Kampffmeyer Food Innovation Study [17], conducted with over 4000 consumers from eight European countries, indicate that food naturalness is a “decisive buying incentive,” and that approximately three quarters of respondents perceived a close “natural” + “health” link. The market research outcomes generally suggest that many consumers in developed countries usually eat natural foods. From the natural science point of view, naturalness definitely does not imply that a food product is healthier, less dangerous or tastier, although this is not how most people perceive naturalness [18,19].

Consumers perceive naturalness as a beneficial characteristic of food items. However, the relative importance of food naturalness varies across lands, cultures and throughout history [20]. Human beings have conventionally sought to monitor and reduce environmental threats. The arrival of increasingly processed foods in developed countries in the 1950s provided longer food shelf life, and better food and nutrition security [4,5]. It was at that time when consumers started showing a strong preference for processed foods. Conversely, consumers’ everyday experience depends on other things apart from these accomplishments. Today, highly globalised markets and intensified food chain production in industrialized economies have added to a knowledge gap and perceived distance between food manufacturers and consumers [6,7].

Globalisation and industrialisation go hand in hand with a more man-made and higher risk that enhance citizens’ perception of modernity risks [21]. In the last few decades, food safety incidents have impacted Europe, such as dioxin and bovine spongiform encephalopathy [22,23]. Consumers are concerned about excess pesticide use by traditional industrial agricultural methods [24], the employment of artificial ingredients, colourants or additives like E133 [25], and questionable food innovations, like genetically modified organisms [26], being introduced. This has made consumers suspicious or sceptical about the negative health consequences that this food system entails [3]. Growing public concern about what the food system does to climate change and its general negative impact on sustainability [27] mean that consumers now question the social and environmental consequences of food production [28,29].

Consumers generally prefer food to be nutrient-fed and satiated, while price and taste are other important factors [30,31]. However, it is often suggested that food consumption in industrialized societies is presently affected by three particular major trends: convenience, health concerns, sustainability [32]. Health concerns are driven by consumers’ affluence, but are also explained by not only the rising number of food- and lifestyle-related diseases (i.e., obesity, diabetes, etc.) [7,33], but also increasing intolerances and allergies to certain components and specific food products like gluten. These aspects also motivate consumers to pay more attention to healthy food items that promote healthier lifestyles at older ages and that lower the incidence of some diseases. Sustainability concerns come over according to increasing knowledge of emissions released by traditional agricultural activities [1]. This has led to an ever-growing expansion of organic farming and markets, and can also explain why some consumers seek products, such as ‘local food’ products (food miles) and why they are willing to pay more for water-saving products [34]. Convenience food refers to the number of meals not eaten at home or home-delivered meals as opposed to homemade meals. This figure has significantly risen in past decades [35], which suggests that consumers are involved in additional capabilities of food items to save time (e.g., frozen foods, ready meals, microwavable food, etc.).

By analysing the factors that impact individual differences in the perceived importance of naturalness, although high mean values of the importance of naturalness in foods (INF) were found in most studies, individual differences appeared in how important naturalness was perceived [8]. For psychological factors, several works have indicated the importance of consumers’ values for explaining INF. Idealism [36], tradition and universalism [37] were positively associated with INF, whereas hedonism and power correlated negatively with INF [37]. Interest shown in health correlated positively with INF [38,39], and attitudes to novel technologies, chemicals and functional foods correlated negatively [38,40,41,42,43,44,45]. Some experiments revealed a certain conceptual similarity between predictors and how INF was calculated. Attitudes to traditional and organic food, along with food involvement and neophobia were positively related to INF in a number of research works [46,47,48,49,50,51]. The research by Olbrich et al. [47], which included over 10,000 German consumers, revealed that attitudes to organic food were related to INF. Similar findings are reported in Taiwan by Hsu et al. [48]. These two experiments indicated that no difference appeared between INF and items assessing organic food preferences. Very few experiments examined the relation linking INF with personality characteristics. Steptoe et al. [39] reported a positive association for INF and the control locus, while Huotilainen et al. [52] found that the perceived INF value was not related to consumer willingness to accept food innovations.

As for consumer attitudes to food naturalness affecting their behaviour and intentions, some studies report INF measurement items overlapping measuring intentions or behaviours in relation to eating organic food [49,51,53,54]. INF influences intention to eat in a more enviro-friendly way [55] with fresh [56], local/traditional [52,57] natural [58] and low-calorie food items [59]. The results cross-country analysis obtained by Hemmerling et al. [57] were inconclusive; INF had a strong impact on local/traditional foods in Italy and Germany, but was negligible in The Netherlands, Poland, France and Switzerland. Only two experiments have investigated how INF affects consumer decisions about eating functional food, but the results were inconclusive. Kraus [60] reported substantial effects in Poland, but the research by Urala and Lähteenmäki in Finland [61] proved unsubstantial. Lähteenmäki et al. [62] also reported that INF adversely affected purchase intention to buy genetically modified cheese in Finland, Denmark, Sweden and Norway. In their works, Lusk et al. [63] saw that INF increased the probability of selecting non-clone or non-hormone milk as opposed to the clone or hormone variety. Many research works undertaken in different countries have revealed that INF significantly affects eating healthy [64,65,66,67,68], organic [69] and traditional foods [46,70], and eating unhealthy [66,67] and convenience foods [71]. Roininen and Tuorila [72] and Zandstra et al. [73] found that INF did not affect unhealthy or healthy eating. Small sample sizes (*n* = 144 and *n* = 132) could potentially explain such insignificant results. With their survey of 197 Spanish consumers, Carrillo et al. [74] found that perceived naturalness of functional foods increased their sales.

Regarding natural food ingredients, which have attracted far more attention from public and food manufacturers, for a few decades now it has been worth emphasising that consumers mainly choose food without additives but, if they are not available, the same consumer chooses food containing natural additives rather than synthetic ones [11,13]. Consumer research has revealed that consumers have increasingly become more knowledgeable of food additives and more frequently prefer natural additives to their artificial analogues [40,75,76]. Unlike artificial sweeteners, which are all capable of structural modification in the hope that better tasting analogues will be discovered, natural sweeteners must be used ‘as are’ simply because any structural change made to a natural sweetener to improve its taste profile will automatically destroy the ‘natural’ proposition and position. So, although the consumer interest level is high, identifying a natural sweetener with the requisite sensory quality is no trivial undertaking [77].

## 3. Natural Sweeteners

Preference for sweet taste is not only innate, but universal [78]. Food products related to a sweet taste characteristically contain simple carbohydrates in the forms of fructose, glucose and sucrose, which are metabolised to produce energy rapidly, as wee as complex carbohydrates in the form of starch for long sustained energy and storage. However, the sweet taste can be induced by the presence of peptides, D-amino acids, glycosides, proteins, coumarins, ureas, substituted aromatic compounds, dihydrochalcones and other nitrogenous substances [79]. Yet all sweet-tasting compounds interact and activate a single receptor, which is expressed on the surface of taste buds, the TAS1R2-TAS1R3 heterodimer, and contains multiple binding sites to explain the range of compounds that induce perceived sweetness [80]. 

Honey used to be the main sweetener in human diet. However, in the 18th century, the process of extracting sucrose from sugar beet and sugar cane grew exponentially and clearly assumed preponderance. Nowadays, sucrose, or common table sugar, remains the most traditionally used sweetener, and is available in a variety of refined forms [81]. In 2018 and 2019, global sucrose consumption came to 174 million metric tons [82]. In the last few years, sugar overconsumption has become pandemic, with serious consequences in public health terms. There is clear evidence for an association between eating too much sugar and being at higher risk for dental caries, type II diabetes obesity and cardiovascular diseases, among other non-communicable diseases [83]. Given this scenario, sweeteners in food products have spread, and this product is major a target of much interest for the industrial and scientific communities.

Many synthetic sweeteners have been developed, but today demand undoubtedly lies in natural sweeteners, preferably the high-intensity kind; i.e., with low-calorie contributions. This trend stems from not only growing consumer concern about the harmful effects of a diet that includes too much sugar, but also the problems that arise from employing artificial food additives. Although many low-calorie sweeteners are readily available, only a few can be used by the food industry, mainly because of safety concerns and technological problems [81]. It is worth noting that, apart from sweetening, these compounds can influence a product’s colour, flavour, texture and shelf life [84].

The most important aspects when selecting a sweetener have to do with its physico-chemical properties, such as thermal stability and solubility in water, but also production cost and safety [85,86]. Its sweetness potency is extremely relevant; that is to say, sweeteners can be classified in line with their intrinsic characteristics (nutritive value, sweetening power) and origin (synthetic, semisynthetic and natural) [87]. Depending on their sweetness level in relation to sucrose, considered an international reference (sweetness potency = 1), sweeteners are grouped into two classes: bulk and intense. Bulk sweeteners have a similar or less sweetening potency vs. sucrose, and are used to typically confer low-calorie food products preservative action, bulk and texture [88]. They can be employed in baked food products, breakfast cereals, preserved foods, desserts, cakes, jams, ice cream and sauces [89]. To the sweeteners in this category we indicate sugar alcohols like maltitol, sorbitol, lactitol, xylitol, erythritol, mannitol, isomalt, hydrogenated starch hydrolysates and hydrogenated glucose syrups [86]. Trehalose and tagatose are two new compounds with a similar suitability to sugar alcohols [86,89]. Bulk sweeteners are frequently utilised in the food industry for the benefits they offer over sucrose in both functional aspects (e.g., lowering the freezing point of a frozen desert or of the Maillard reaction) and nutritional terms (e.g., slower assimilation). However, these sweeteners do not substantially lower the calorie value of a food [89].

The sweetness that intense sweeteners provide is much more than sucrose and in different potencies [88]. Given their high sweetness potency, very small amounts are required to accomplish the desired sweetening effect. Hence their contribution to a product’s energy value is minimum, which is most advantageous [87]. Despite them often being called “artificial sweeteners”, such compounds can be synthetic (e.g., saccharin, aspartame, sucralose, acesulfame-potassium, cyclamate, alitame, neotame, dulcin), semisynthetic (e.g., neohesperidine dihydrochalcone) or natural (e.g., rebaudioside and stevioside) [88]. Intense sweeteners are of widespread use in processed foods, especially carbonated and non-carbonated drinks, canned food, baked food, sweets, jellies and puddings [89].

Although an origin-based sweetener classification is not considered by authorities like the European Food Safety Authority (EFSA) or the US Food and Drug Administration (FDA), we adopt this classification because, given their growing popularity, we pay attention to natural sweeteners.

Natural sweeteners encompass wide-ranging compounds like sugars, sugar alcohols, amino acids, proteins, terpenoid glycosides and some polyphenols [84,85]. Having said that, only those that possess relevant characteristics, e.g., safety, good taste, high stability, good solubility and reasonable cost, are found on the market as widely used sweeteners [11,90]. The present review focuses on the natural sweeteners that comply with these principles.

### 3.1. Sugars

The relative sweetness potency of carbohydrates is consistently lower than that of sucrose (a reference compound), except for fructose, which is the sweetest natural sugar (relative sweetness = 1.43) which is abundance in fruit, agave nectar, honey and some vegetables [85]. Fructose and glucose and are the two most widely adopted monosaccharides as natural sweeteners by the food industry [85]. Fructose replaces sucrose in a range of food products thanks to its lower glycaemic index, sweetening strength and low cost, and its ability to improve overall end product quality characteristics; flavour, colour texture, and shelf-life stability [84]. High fructose syrups are widely employed, mostly those produced with corn starch by an elaborate technological process, and given their interesting texturising ability, flavour profile [85]. When ingested as large amounts, malabsorption and consequent gastrointestinal disturbances may occur, and excessive intake may lead to metabolic changes; e.g., insulin resistance, high plasma triglycerides, etc. [84,91].

In this context it is worth highlighting two new compounds: trehalose and tagatose. They have been relatively recently approved as novel food ingredients or novel foods and appear on the market as sucrose alternatives [86]. The trehalose disaccharide comprises two glucose units linked by an α-1,1-glucosidic bond and occurs naturally in plants, fungi, insects, algae bacteria and yeasts [86]. The commercial product is acquired from starch by following an enzymatic process [92] and it has a relative sweetness power of 0.43 [85]. Trehalose is well appeciated because it induces a low glycaemic response and helps to maintain dehydrated and frozen food products fresh via the stabilisation of colour, texture and flavour [86]. It reduces starch retrodegradation and does not participate in Maillard reactions. It is an ingredient frequently found in sports drinks and health bars [93]. Tagatose is a fructose isomer found naturally-occurring in certain fruit and dairy products [90]. It is considered a prebiotic and a flavour enhancer [11]. In industrial terms, it is produced from lactose following a multistep enzymatic process, plus fractionation and purification [14]. Its sweetness potency comes close to that of sucrose, 0.92, with the advantage of being differently metabolised by contributing to fewer calories, evoking a weaker glycaemic response [86,87,90] and does not favour dental caries. As it is only partly digested, tagatose can bring about diarrhoea, abdominal discomfort and flatulence when ingested as high doses [86]. Tagatose is used to prepare energy bars, breakfast cereals, chocolate gums, caramel, ice cream, soft drinks and yogurt [11,90].

### 3.2. Sugar Alcohols

Sugar alcohols, or polyols, are low-digestible carbohydrates that occur naturally in fruit, vegetables, mushrooms and algae [94], and have been employed as an alternative type of sweetener in recent years. The sugar alcohols allowed by the food industry to be used as nutritive or bulk sweeteners include maltitol, mannitol, sorbitol, xylitol, erythritol, isomalt and lactitol. Some other relevant compounds that enter this category are arabitol and hydrogenated starch hydrolysates, despite not being permitted in the EU [87]. They are obtained generally by catalytic hydrogenation from the corresponding aldose sugars [84]. For certain sugar alcohols, like erythritol, methods based on fermentation or enzymatic conversion with osmophilic yeasts or fungi have been followed [11]. By the way, mannitol, sorbitol and maltitol are easily extracted from brown algae (i.e., *Laminaria* species) [95]. Sugar alcohols are frequently employed for food product reformulation purposes where, other than sweetness, texture and the bulk of sugar play a key role in sugar-free cookies, cakes, sweets, chocolate and gums [86,96]. They are applied to pharmaceutical products like throat lozenges [86]. Polyols offer two major advantages over sugar as food ingredients: (1) do not favour tooth decay because they are not fermentable by oral bacteria; (2) lower calorie content and glycaemic index, which are most interesting for diabetics [86]. Sugar alcohols also present prebiotic properties and, like fibres, contribute to healthy intestinal microbiota [97]. Sugar alcohols are universally considered safe and have no established acceptable daily intake (ADI), but should be used in line with good manufacturing practices (GMP) [84,94].

Sugar alcohols are frequently used together with intense sweeteners to mask off-flavours of the latter, while conferring the bulkiness that low-calorie sweeteners cannot [84,98]. Unlike sugars, they are not subject to Maillard reactions and leave a cooling effect in the mouth, which could be desirable for certain products, but particularly in many others (e.g., baked goods) [94]. Overall, of all the allowed sugar alcohols, the properties of xylitol, erythritol and maltitol come the closest to those of sucrose, with a relative sweetness of 0.63, 0.87 and 0.97, respectively. This is why they are the most widely used [85,99]. Unlike sucrose and glucose, sugar alcohols are not totally digested, which is why excess ingestion can lead to gastrointestinal symptoms even in healthy people, like laxative side effects, so consumers with inflammatory bowel disease should be very careful with them [97]. Notwithstanding, frequent intake seems to result in better tolerance [99]. Besides the above-cited side effect, they display no other health-related problems in association with high-potency artificial sweeteners [10]. As with all sweeteners, the safety of sugar alcohols is currently being reassessed by EFSA and new data are expected to become available at the end of 2020 [100].

### 3.3. Terpenoid Glycosides

Steviol glycosides are a group of sweet compounds which are extracted from the leaves of a plant native to South America called *Stevia rebaudiana* Bertoni (Asteraceae), which is currently grown in some countries in Europe and Asia. Typically, the above-mentioned glycosides represent up to 15% of the dry matter in plant leaves. Ten main ent-kaurane diterpenoid glycosides exist, and they all have the same steviol core structure. Stevioside, followed by rebaudioside A, are the two most abundant and commercially relevant ones [90]. They leave a very sweet taste in the mouth that is hundreds of times superior to sucrose, which makes them very interesting sweeteners [85]. Rebaudioside A, whose relative sweetness is 250–450, is the most appealing steviol glycoside, and offers a taste like sucrose with no off-tastes, while stevioside has a slight bitter side effect [85,101]. Seeing as the leaves of the plant cannot be utilised directly in the USA and EU, steviol glycosides are extracted with water before being redissolved and recrystallixed from a hydro-alcoholic solution [87]. Steviol glycosides are hydrolysed to steviol by colonic microbiota. Most steviol is absorbed by the intestine before reaching the liver, where it goes through a process of conjugation with glucuronic acid to produce steviol glucoronides, which are finally excreted mostly in urine [102]. Consuming steviol glycosides is safe provided it lies within the 4 mg/kg of body weight/day limit [103]. Both their calorie contribution is non-significant, so they are suitable for diabetic patients. They have also been attributed anti-inflammatory and immunomodulatory diuretic and anti-hypertensive properties [104]. As for their physico-chemical characteristics, they remain moderately stable at high temperature and may be used within a pH range from 2 to 10 [87]. Steviol glycosides have been widely used to produce confectionery, chocolates, baked goods, yoghurts, ice cream, gums, sauces, jam dairy products and drinks [11,105].

Another interesting sweetening compound is glycyrrhizin, or glycyrrhizic acid, which is isolated from *Glycyrrhiza glabra* L. roots (Fabaceae), which is a liquorice plant [90]. This molecule offers a relative sweetness of 90 [85]. Its use as a sweetener is permitted in Japan and other countries, but not in the USA and EU [85], where glycyrrhizin is approved only as a surfactant and flavouring agent, and also in the form of ammoniated glycyrrhizin, which is considered Generally Recognised as Safe (GRAS) [106]. Glycyrrhizin intake should never exceed 100 mg/day, considering all its sources in the diet, given the risk of toxic effects: hypertension and hypokalaemia-induced secondary disorders [107,108]. Some authors indicate that glycyrrhizin could have beneficial effects on intestinal microbiota [97]. The applications of glycyrrhizin as foaming agent and flavour enhancer include baked goods, ice cream, confectionery, gums and beverages [99].

### 3.4. Proteins

Sweet-tasting proteins are naturally-occurring in some exotic plants, and their sweetness is hundreds to thousands of times superior to sucrose [85]. Thaumatin comprises a mixture of six closely interrelated proteins, thaumatin I, II, III, a, b and c, extracted from *Thaumatococcus daniellii* Benth fruit (Marantaceae), which is native to western Africa. Thaumatin I and II are the main forms, despite all isoforms being sweet-tasting [90]. No unanimous value for its sweetening potency exists, but it is estimated to be about 1600–3000-fold higher than sucrose [11,85]. Extraction is performed by water and mechanical methods [87]. Current thaumatin production does not meet demand, and alternative methods to produce it through microorganisms and transgenic plants are growing [109]. Thaumatin is permitted in both the EU and the USA, where it is GRAS [97]. Owing to lack of toxicity, its ADI is still to be established [110]. There is, however, a risk of allergic reactions [111]. The metabolism of thaumatin is similar to that of any other protein in human diet. Its energy input is 4 kcal/g, which is negligible as minor amounts are used in practice [101]. The main problems with its use are late onset of action and a slight liquorice off-taste, which may interfere with consumer acceptance. Hence its use in large amounts is not recommended. Nevertheless, it works extremely well when employed in conjunction with other sweeteners to diminish bitterness and confer foods an umami flavour [90]. As regards physico-chemical properties, it is highly soluble in water, and well resists high temperature and acidic pH [87]. Thaumatin is frequently employed in processed vegetables, sauces, soups, poultry, products deriving from egg, gums and fruit juice [87].

Several other sweet proteins are known, with the most promising ones being brazzein, mabinlin, monelin, miraculin, pentadine, curculin (neoculin) and lysozyme, but more studies are necessary to ensure their safety and applicability [90].

## 4. Production of Safe Enviro-Friendly Natural Sweeteners

Natural sweetener production must remain safe with no adverse environmental consequences. It is presently necessary to guarantee that our food system does not pose health problems to consumers and our planet, as reflected in the recent ‘EU green deal’ [112]. This section summarises the production of the following sweeteners, which are highlighted in preceding sections given their relevance for industrial food processing.

### 4.1. Erythritol

Industrial erythritol production has gained prominence with the rapid development of the electrochemical process. During this process, erythrose and erythritol are produced by the electrolytic decarboxylation of arabinoic or ribonic acid. The substrates for the reaction are obtained by the decarboxylation of C-6 sugars [113]. However, a more natural method involves the biotechnological process, which results in higher yields from fermenting a sugar source. Erythritol derives from fermentation processes, conducted mostly by fungi or synthesized by lactic acid bacteria. In order to produce erythritol, the common pathways among east-like fungi genera include: *Trigonopsis*, *Candida*, *Pichia*, *Moniliella*, *Yarrowia*, *Pseudozyma*, *Trichosporonoides*, *Aureobasidium*, *Trichoderma* [114]. For industrial production purposes, *Yarrowia lipolytica*, *Moniliella pollinis* and *Trichosporonoides megachiliensis* are reported as effective [113]. One main part of the production process involves separation and purification steps because they are crucial when erythritol is taken as a food additive. A patent describes that to recover erythritol from the culture medium, separation from fermenting microorganisms is required, followed by ion exchange chromatography and crystallization. Moreover, a chromatographic separation step was subject to activated-carbon treatment in order to recover the erythritol fraction [115].

### 4.2. Tagatose

As a natural sweetener, biotechnological tagatose production by enzymatic isomerisation is a preferred alternative to chemical processes. For biological D-tagatose manufacturing, several biocatalyst sources can be resorted to; e.g., L-arabinose isomerase (l-AI) EC 5.3.1.4, which can catalyse the conversion of D-galactose into D-tagatose, and also for converting L-arabinose into L-ribulose, due to the similar configurations of substrates [116,117] yet biological D-tagatose production is limited given the less bioconversion efficiency of l-AI, a metal ion requirement, and the poor thermostability and low affinity of the enzyme for D-galactose. It has, thus, been suggested that applying protein engineering and genomic tools may enhance the bioconversion efficiency for D-tagatose production by amending the functional properties of l-AI [118]. Applying high-throughput screening or a selection method helps to evaluate individual protein variants and, hence, increase the possibility of screening specific mutants with greater catalytic activity. During D-tagatose production, the safety problem caused by enzyme or cells of not GRAS hosts can be overcome by transferring the gene of L-arabinose isomerase to GRAS hosts like *C. Glutamicum*, *Corynebacterium ammonagenes* and *Bacillus megaterium* [119]. Ultimately, more research needs to be conducted to explore new sources of biocatalysts from GRAS microorganisms, apart from enzyme secretion and expression in a food-grade microbial host.

### 4.3. Steviol Glycosides

The raw materials employed in the manufacturing process of Steviol glycosides preparations are crushed leaves from the perennial shrub *Stevia rebaudiana* (Bertoni) Bertoni of the family Asteraceae (Compositae). The literature indicates several alcohols and ion exchange resins used during the manufacturing process [120]. Extracting glycosides from stevia leaves involves thermal extraction and maceration. Both the quality and yield of the extracted products can increase by following techniques like supercritical fluids, ultrasonic waves and microwaving [121]. Besides, a multistage membrane process, which has been developed to concentrate glycoside sweeteners, is also highlighted in the report, with bitter-tasting components from the sweetener concentrate being washed out during the nanofiltration process.

The conventional extraction processes described in the literature often follow a similar methodology, whereby stevia leaves are extracted with hot water or alcohols. In certain cases, leaves are pre-treated with non-polar solvents (e.g., hexane or chloroform hexane) to eliminate lipids, essential oils, chlorophyll and other non-polar substances. With this pre-treatment, extracts are clarified by precipitation with either salt or alkaline solutions, and then finally concentrated and redissolved in methanol for the crystallisation of glycosides [122]. Other extraction procedure steps involved are described in [123], where stevia leaves were soaked in warm water to dissolve glycosides before the precipitation and filtration of the resultant solution, followed by concentration by evaporation, ion exchange purification, spray drying and crystallisation to produce a white powder and crystals. Rao et al. [124] applied ultra- and nano-filtration membranes to develop a simple eco-friendly and low-cost process to isolate steviol glycosides, which resulted in the final product’s improved taste profile.

### 4.4. Glycyrrhizin

The methods followed to prepare glycyrrhizic acid (GA) from liquorice roots have been investigated by several researchers. The literature reports a number of procedures as regards solvent extraction by various organic solvents, purification by ion exchange and polymeric resins, chromatographic separation, adsorption, foam separation, supercritical fluid extraction, microwave-assisted extraction (MAE) and multistage counter-current extraction (MCE) to extract GA [125]. Most existing processes to extract and purify the sweet ingredients from liquorice roots involve several steps and large quantities of solvents and chemicals. Extracting GA from roots includes extraction with hot water at ambient pressure in the presence of a number of additives, such as alkalis, as well as mineral acids, ethyl alcohol like aqueous ammonia, methanol and ethanol, which are the most well-accepted technologies. The primary aqueous extract from liquorice roots contains GA and many other water-soluble substances, which are then subjected to further process more purified products. Pure GA is also prepared from liquorice roots using alcohol as the extraction solvent in an ultrasonic device, followed by purification [126]. The conventional solvent extraction technique followed to extract GA from liquorice offers several disadvantages, namely considerable solvent requirements, longer extraction time, lower yields and higher extraction temperature. All this requires developing an effective economical extraction method [126]. The purification procedure involves the acidification of the extract by adding acids like H_2_SO_4_ or HCl acids to form the solid product of GA salt (at pH 1–2). Ultrasound assisted extraction has shown that the extraction rate rises due to cavitation because the developed cavity grows in size and then abruptly collapses with the release of energy at an enormous rate, which thus increases the local temperature and pressure [127]. Therefore, greater solvent penetration in cellular materials takes place, which improves the cell content release in the bulk medium [127].

### 4.5. Thaumatin

The thaumatin production process can be strongly affected depending on the quality and availability of source materials [128]. In order to achieve stabler protein production that meets its demand, a series of studies were conducted, which involved thaumatin production with genetically engineered microorganisms and transgenic plants (see the studies by [129,130]).

Although using a plant system offers some advantages over microbial systems in terms of its scalability, safety and economy, they still lack some benefits that can be obtained from microbial hosts, such as the possibility of controling growth conditions and product consistencies from batch to batch [128]. Biochemical production methods have been considered because the natural production of these proteins is normally too expensive. Recombinant DNA technology is applied to produce sweet proteins in a host organism. The most promising host known is the methylotrophic yeast *Pichia pastoris*. This yeast has a tight regulated methanol-induced promoter that well controls recombinant protein production [128]. Despite thaumatin having been studied by several researchers in the last 30 years, there is still much to be done to improve its production by biochemical routes. As the literature evidences, biological products are emerging as a promising applicant in the food industry, hence the huge potential for future research to centre on using advanced computational techniques to optimise thaumatin bioproduction.

Other natural sweeteners with enviro-friendly production methods that are becoming popular food ingredients for health-conscious consumers are briefly described below:

(1) Raw Honey: one of the oldest natural sweeteners. Honey is sweeter than sugar, and is the only sweetener obtained from an animal source (insect bees, minilivestock). Honey is a sugar secretion that is deposited on honeycombs by bees *Apis mellifera*, *Apis indica* (Indian Bee), *Apis dorsata* (Rock Bee), among other Apis species of the family Apidae [131].
(2) Blackstrap Molasses: the by product from raw sugar refinery or a sugarcane factory; it is the thick dark, viscous liquid that is left after the final sugar crystallisation stage from which no more sugar can be crystallised economically by usual methods [132].
(3) Real Maple Syrup: it is made from the sap exuded from stems of the genus *Acer*, usually in spring. Sap primarily contains water and sucrose, with varying amounts of amino and organic acids and phenolic substances, which is concentrated by heating to produce a wide range of flavour compounds [133].
(4) Coconut Sugar: it is locally produced from the phloem sap of coconut palm tree (*Cocos nucifera* L.) blossom. Juice collectors climb palm trees and cut off unopened inflorescences with sickles. The escaping sap is collected in bamboo or plastic containers for 8–12 h. Lime is sometimes added to prevent sap from fermenting [132].
(5) Other combinations: they involve production that blends several natural product sweeteners, such as a low concentration of steviol glycosides (<0.5 percent per dry leaf weight) with a small amount of raw organic sugar cane. Similarly, a combination of sweetening solutions, e.g., Erysweet+, a stevia erythritol blend, and KetoseSweet+, an allulose, stevia and monk fruit blends are becoming popular beverages [134].

Consumers are eager to purchase products with natural ingredients and clean labels, preferably with further functional properties, but which do not compromise taste. In order to achieve this trend, food industries are now willing to reformulate their food products to include alternative natural sweeteners to sugar.

## 5. Health Impacts

For natural sweeteners are deemed suitable to be extensively used and marketed, they must be safe, offer good flavour with a high degree of solubility and a good level of stability, and offer reasonable cost-effective applications [135]. This paper only investigates the natural sweeteners that meet all these criteria [11] in relation to their health impacts.

The two major compounds of bulk sweeteners are erythritol and tagatose. Erythritol is allowed in both the USA and the EU, but there are restrictions on use in drinks with the latter. As a bulk sweetener, it has approximately 65% of sucrose sweetness, but it does not lead to tooth decay and is neither toxic nor carcinogenic for the amounts added to food. The main products for which erythritol is employed are baked goods, frostings, coatings, chocolate, fermented milk, low-calorie beverages, chewing gums, sweets, among others [129,135]. In 2014, a scientific panel, mandated by EFSA, ruled out its laxative properties and declared it safe for use without defining its acceptable daily intake (ADI) [136]. Based on acute toxicity investigations, and following oral administration, erythritol is graded as being essentially non-toxic. Subchronic research further enhances erythritol’s safety. Chronic research (up to 2 years) has demonstrated that erythritol has no effect on either survival or carcinogenicity [137,138]. At high doses (up to 16 g/kg body weight), erythritol affects neither the reproductive capacity nor fertility of parental rats. No adverse effects have been observed on developing foetuses [137,138,139,140]. Erythritol has no mutagenic potential, as observed in the Ames and chromosomal aberration tests [137,138,141,142]. Animal toxicity tests and human clinical trials have reliably shown that erythritol is safe. Erythritol has never been predicted to have adverse effects when applied for its intended use in food [137,138].

Erythritol has been found to reduce the risk of caries in several trials [143,144,145,146,147]. As erythritol does not affect insulin levels or glucose, it is an appropriate sugar substitute in diabetes patients, and also for individuals who wish or need to regulate their blood sugar levels because of prediabetes or compromised carbohydrate metabolism [148,149]. Diabetes patients can benefit from the vascular effects of erythritol, as mentioned above. It is assumed that endothelium is not compromised by erythritol in non-diabetic subjects, but in diabetic subjects where endothelium is under diabetic stress, erythritol can transfer a range of damage and dysfunction parameters to a safer side, as *in vitro*, *ex vivo* and *in vivo* studies report [148,150,151]. Erythritol can also be regarded as a substance with a beneficial impact on the endothelium under high-glucose conditions by contributing to avoid or delay the onset of diabetic complications [152]. The erythritol attribute has minor effects on several targets and can also prove beneficial. A compound with a strong biological effect is not as appropriate for chronic supplementation as required in diabetes. The option would be to use a substance like erythritol with moderate protective effects. Erythritol is not only valuable, but should be considered a recommended sugar replacement for the rapidly increasing numbers of people with diabetes or prediabetes to reduce their chances of developing diabetic complications [152,153].

Tagatose comes in very small amounts in fruit and heat-treated dairy products. Its potency vs. sucrose is 92 %, which means that it comes close in taste, but only adds 1.5 kcal/g, which makes it safe for diabetics to use without harming teeth. Tagatose is approved in the US as a GRAS compound, is permitted in the EU as a food ingredient and in many other countries with practically no toxicity associated with its use. Tagatose uses in the food industry include yoghurts, frostings, cereals, beverages, chewing gum, fudge, caramel, fondant, chocolate and ice cream [129,135,154].

Tagatose’s safety and toxicity dimensions have been explored in animal and human subjects [118]. As tagatose intake increases above 10%, adverse reactions (increased liver weight and hypertrophy) have been reported in rats [155]. Consequently, the 5% tagatose level is a known safe dose that has no side effects. at reproductive performance is not impaired, even when tagatose intake is as high as 20 g/kg body weight/day [156]. Human clinical experiments to study D-tagatose use have been based mainly on its gastrointestinal and urecaemic consequences. High plasma uric acid levels are associated with purine metabolism disorder and gout development. A significant rise in the plasma uric acid concentration occurs in both the healthy and non-insulin-dependent diabetes mellitus populations after a single oral 75 g dose of D-tagatose [157]. A lower D-tagatose dose (45 g/day; 15 g, 3 times/day [TID]) is considered safe for healthy human subjects because it has no adverse effects on glycogen levels, plasma uric acid, and liver function [158]. An intake of 45 g D-tagatose/day (15 g TID) for 1 year does not induce any adverse effects on plasma uric acid levels in patients with non-insulin-dependent diabetes mellitus [159]. The above D-tagatose dose also tends to reduce postprandial plasma glucose levels. However, very few records suggest any gastrointestinal problems (nauseas, mild to severe flatulence and diarrhoea) following the intake of 30 g of D-tagatose as a single dose [160]. Given the above considerations, the “No Observed Adverse Effect Level” (NOAEL) for tagatose is set at 45 g/day or 0.75 g/kg body weight/day [161].

Regarding high-potency sweeteners, steviol glycosides (E 960) [162] are a good example of natural compounds disseminated widely worldwide. Steviosides have been used in large quantities in Japan for more than 20 years and have no documented side effects. Stevia safety is also responsible mostly for the low-absorption steviol glycosides in both humans and rats in stomach and upper intestine [121].

The use and safety of steviol glycosides has been reviewed and evaluated worldwide by a range of scientific bodies and regulatory organisation. High-purity extracts of stevia leaves have been approved for use in food and beverages by over 150 countries and regions [163]. During its 69th meeting, the Joint FAO/WHO Expert Committee on Food Additives (JECFA) set an ADI of 4 mg/kg bw/day for steviol glycosides in 2008, expressed as steviol equivalents. JECFA reaffirmed this ADI during its 82nd meeting in 2016 [164]. The Food Standards Australia New Zealand [165] and EFSA [166] have defined an ADI of 4 mg/kg bw/day for steviol glycosides (expressed as steviol equivalents). Stevia mutagenicity has been studied in many trials, although they gave contradictory results. For example, two studies concluded that, in certain assays, stevia demonstrated a dose-dependent mutagenic effect, but the same studies also concluded that stevioside is non-mutagenic [167,168]. Several other findings indicate that the plant lacks mutagenic effects [169,170]. Despite reports not being harmonious, the FDA continues to monitor this herb as a sugar replacement, while other findings reveal that steviol and stevioside do not interfere with DNA and have no genotoxicity [171]. Mizushina et al. [172] suggested that stevioside is not involved in bladder carcinogenesis. Up to 2500 mg/kg body weight/day has been safely used in rats and enabled their normal growth and reproduction [173]. After 14 consecutive days of administering steviosides as part of acute toxicity trials, no histopathologicity, no lethality and no morphological modifications were recorded in rodents [174]. In another study, the oral administration of an aqueous extract taken from stevia leaves (up to 10%) revealed no adverse effects on female rat fertility and no teratogenic effects [175]. It has also been shown that both stevia and stevioside are safe when used as sweeteners. This is appropriate for diabetic and phenylketonuric patients, and also for obese individuals who wish to lose weight and to remove sucrose from their diet. After intake, no allergic reactions or toxicity were reported [176]. In the long term, randomised, double-blind, placebo-controlled trials indicate using steviol glycosides as a sweetener with no toxic effects for humans [177]. Stevia’s safety has also been confirmed by recent studies, which demonstrated that steviol glycosides are not mutagenic, carcinogenic or teratogenic, they and do not cause toxicity [178,179]. Recently, following oral administration, a toxicological stevia leaf ethanolic extract evaluation revealed no harmful effects on subchronic oral toxicity and genotoxicity. The authors proposed that stevia leaves have the potential to be considered functional food and a nutritional supplement, rather than sweetener [180].

Another high-potency sweetener is glycyrrhizin (E 958) [181]. This compound, which is also known as glycyrrhizic acid, can act as a sweetener with a potency 50-fold sweeter than sucrose, but is also employed as a foaming agent and a flavour enhancer. This substance is legally used in both the US and EU as mono-ammonium glycyrrhizinate and ammoniated glycyrrhizin. Glycyrrhizin has antiviral, anticancer antioxidant, anti-inflammatory and hepatoprotective effects [97], but also has potential hypertensive effects and an intense aftertaste [182]. In the gut, glycyrrhizin is de-glycosylated to glycyrrhetic acid (a major product) by *Eubacterium* spp. *Bacteroides* J-37 and to 18β glycyrrhetic acid 3-*O*-monoglucuronide (the minor product) by *Bacteroides* J-37 and *Streptococcus* LJ-22. *Eubacterium* spp. can be used to convert 18β-glycyrrhetic acid 3-*O*-monoglucuronide into glycyrrhetic acid [182,183,184]. These glycyrrhizin metabolites (particularly 18β-glycyrrhetinic acid) are significant anti-tumour cytotoxic agents with potent inhibitory effects on anti-platelet aggregation activity and rotavirus infection [185]. Some results indicate that the glycyrrhizin/intestinal microbiota interaction has beneficial effects on hosts [183,184,186].

Thaumatin (E 957), a mixture of five proteins (thaumatin I, I, III, a, b), is also employed as a sweetener in many countries. If we consider its health effects, thaumatin does not induce tooth decay and is suitable for diabetics, as opposed to artificial sweeteners [187]. The metabolism of this sweetener is the equivalent to other dietary proteins. The research work by Hsu et al. [188] demonstrates that thaumatin is digested more quickly than egg albumin. Moreover, several studies addressing thaumatin safety aspects indicate that this sweetener induces neither toxicity nor allergenicity [128]. Some studies have evaluated thaumatin toxicity; e.g., the Joint FAO/WHO Expert Committee on Food Additives, Food and Agriculture Organization of the United Nations and World Health Organization [189] study reveals that protein is void of toxic, genotoxic or teratogenic effects. Several studies offer compelling evidence that thaumatin is not an allergen to either oral mucosa or other treatment-associated allergic effects [128]. Higginbotham et al. [190] also state that thaumatin has no harmful impact when employed as a flavour additive or a partial sweetener at a particular intake level. This protein’s safety has been evaluated by the Scientific Committee for Food of the European Commission (SCF) and JECFA, which concluded that it should be listed as an acceptable ingredient [110]. This sweet protein has been approved in the European Union since 1984 (E957) according to Annex II of Regulation (EC) No. 1333/2008 [110] and maintains its GRAS status in the USA. It was licensed for use in pharmaceuticals and food in the UK in 1983, except for baby food. It is an approved high-intensity sweetener and flavour enhancer in most other countries [191]. The Panel on Additives and Products or Substances used in Animal Feed (FEEDAP) [192] also indicates the safety of this protein in animals and its use is permitted as an additive from 1 to 5 mg/kg. Thaumatin is also employed as a sweetener in some foodstuffs like ice cream and sweets at the permitted 50 mg/kg dose. In dairy products and soft drinks, it is primarily utilised as a flavour enhancer within the range from 0.5 mg/L and 5 mg/kg [89].

There are a few other natural sweeteners that can be used in the future, but are not actually found in foodstuffs. Some examples of these substances are brazzein and monatin, which is attributable to their rarity and low yield when isolated from plant matrices.

Table 1 lists the main attributes of natural sweeteners for their use by taking into account health impacts.

## 6. Conclusions

Society is becoming increasingly aware of the utmost importance of eating a balanced diet to maintain and promote health. Excess sugar consumption is now a cross-cutting concern, but this habit is not an easy one to break, so sugar-free or low-sugar foods and drinks are in great demand and the sweetening agents that make them feasible are high-value ingredients. Today the food industry applies bulk and intense sweeteners, which are mainly synthetic in origin, to substitute sugar (sucrose). Consumers are all the more eager to eat products with natural ingredients and clean labels, preferably with other functional properties, and that do not compromise taste. To achieve this trend, the food industry now has alternative natural sweeteners at its disposal, like high-fructose corn syrup, sugar alcohols (polyols) and, quite recently, steviol glycosides tagatose and thaumatin, which offer consumers the advantage of additional health benefits. Nature is an incredible source of valuable compounds, including those with a sweet taste, of which many have not yet been explored. Nevertheless, it must be emphasised that being natural does not ensure their success on the market. It should also be noted that a long traditional use in some restricted societies and areas around the globe, and this despite providing some reassurance, cannot rule out the need to conduct detailed scientific studies to prove the safety of the natural compounds to be used as food additives and, for example, as sweeteners. The food industry needs to face the challenge of developing new products with natural functional sweeteners to continue innovating and satisfying consumers. Finally, although compounds like glycyrrhizin, an approved flavour enhancer, are not used as a sweetener, can play a relevant role in improving product characteristics, such as flavour, and need to be considered by industry.

## Figures and Tables

**Table 1:** The main attributes of natural sweeteners for their use by taking into account health impacts.

Natural Sweetener	Attribute(s) and Reference(s)
Erythritol	Non-carcinogenic [137,138]; Non-mutagenic [137,138,141,142]; does not affect glucose or insulin levels [148,149]; beneficially impacts the endothelium [152]
Tagatose	Lowers postprandial plasma glucose levels [160]
Steviol glycosides	Non-genotoxic [171]; non-carcinogenic [172,178,179]; non-allergic [176]; non-teratogenic and non-mutagenic [178,179]
Glycyrrhizin	Anticancer, antiviral, antioxidant, anti-inflammatory, and hepatoprotective [97,185]
Thaumatin	Does not induce tooth decay [187]; not toxic and non-allergic [128]
